# Consequences of past climate change and recent human persecution on mitogenomic diversity in the arctic fox

**DOI:** 10.1098/rstb.2019.0212

**Published:** 2019-11-04

**Authors:** Petter Larsson, Johanna von Seth, Ingerid J. Hagen, Anders Götherström, Semyon Androsov, Mietje Germonpré, Nora Bergfeldt, Sergey Fedorov, Nina E. Eide, Natalia Sokolova, Dominique Berteaux, Anders Angerbjörn, Øystein Flagstad, Valeri Plotnikov, Karin Norén, David Díez-del-Molino, Nicolas Dussex, David W. G. Stanton, Love Dalén

**Affiliations:** 1 Department of Bioinformatics and Genetics, Swedish Museum of Natural History, Stockholm, Sweden; 2 Department of Zoology, Stockholm University, Stockholm, Sweden; 3 Department of Archaeology and Classical Studies, Stockholm University, Stockholm, Sweden; 4 Norwegian Institute for Nature Research, Trondheim, Norway; 5 Museum ‘Severnyi Mir’, Yakutsk, Russia; 6 Operational Direction ‘Earth and History of Life’, Royal Belgian Institute of Natural Sciences, Brussels, Belgium; 7 Mammoth Museum of Institute of Applied Ecology of the North, North-Eastern Federal University, Yakutsk, Republic Sakha (Yakutia), Russia; 8 Arctic Research Station of Institute of Plant and Animal Ecology, Ural Branch of Russian Academy of Sciences, Yamal-Nenets Autonomous District, Russia; 9 Arctic Research Center of Yamal-Nenets Autonomous District, Salekhard, Russia; 10 Canada Research Chair on Northern Biodiversity and Centre for Northern Studies, Université du Québec à Rimouski, Rimouski, Canada; 11 Academy of Sciences of Sakha Republic, Lenin Avenue 33, Republic of Sakha, Yakutia, Russia

**Keywords:** mitochondrial DNA, mitogenome, arctic fox, bottleneck, climate change

## Abstract

Ancient DNA provides a powerful means to investigate the timing, rate and extent of population declines caused by extrinsic factors, such as past climate change and human activities. One species probably affected by both these factors is the arctic fox, which had a large distribution during the last glaciation that subsequently contracted at the start of the Holocene. More recently, the arctic fox population in Scandinavia went through a demographic bottleneck owing to human persecution. To investigate the consequences of these processes, we generated mitogenome sequences from a temporal dataset comprising Pleistocene, historical and modern arctic fox samples. We found no evidence that Pleistocene populations in mid-latitude Europe or Russia contributed to the present-day gene pool of the Scandinavian population, suggesting that postglacial climate warming led to local population extinctions. Furthermore, during the twentieth-century bottleneck in Scandinavia, at least half of the mitogenome haplotypes were lost, consistent with a 20-fold reduction in female effective population size. In conclusion, these results suggest that the arctic fox in mainland Western Europe has lost genetic diversity as a result of both past climate change and human persecution. Consequently, it might be particularly vulnerable to the future challenges posed by climate change.

This article is part of a discussion meeting issue ‘The past is a foreign country: how much can the fossil record actually inform conservation?’

## 1. Introduction

The field of conservation biology can be divided into two major paradigms [1]. One of these, the small population paradigm, is concerned with the threats posed by small population size itself, such as genetic and demographic stochasticity. The second, termed the declining population paradigm, describes the extrinsic processes that cause small populations to become small in the first place. Ancient DNA has emerged as an increasingly popular tool to study both these paradigms. For example, the use of a temporal approach can enable quantification of changes in adaptive potential, as well as inbreeding levels and genetic load, in small populations [2]. In addition, the recovery of DNA from ancient remains makes it possible to sample across known past environmental changes, and consequently to identify factors that have led to population declines in wild populations [3,4].

The main external processes that are thought to cause population declines include overharvesting, habitat destruction, competition from invasive species, and climate change. In many species, it is likely that several of these processes have contributed to population declines, either simultaneously or one after the other. For example, the present-day population sizes in most Arctic species are likely the result of an interaction between prehistoric changes in climate and more recent anthropogenic impacts.

For Arctic species, the end of the Pleistocene was a period of dramatic shifts in habitat availability, which resulted in losses of genetic diversity and population replacements [5–7]. In general, the warming climate at the onset of the Holocene led to decreases in geographical distributions of Arctic taxa. However, it has been proposed that the underlying processes for these range contractions differed among species, either being characterized by population movements (habitat tracking; [8,9]) or local population extinctions [10]. In more recent times, human activities such as habitat destruction, pollution and hunting have contributed to local population decreases and extinctions [11], including in Arctic species. Especially during the last 100 years, these have led to demographic bottlenecks and ensuing losses in genetic variation [12].

One species that both contracted in range at the end of the last glaciation and has been affected by more recent human activities is the arctic fox (*Vulpes lagopus*). The arctic fox had a wide distribution during the last glaciation [13]. Today, however, the arctic fox is restricted to a circumpolar distribution along the northern fringe of continental Eurasia and North America, as well as on high-latitude islands such as Greenland, Iceland and Svalbard [14]. On a worldwide scale, the arctic fox is classified by the International Union for Conservation of Nature as Least Concern, but the population in Scandinavia is classified as Endangered in Regional Red List assessments. The population started declining owing to a severe demographic bottleneck at the beginning of the twentieth century [15]. Heavy hunting pressure fuelled by a profitable fur trade led to a decrease from several thousand to only a few hundred individuals [16,17]. Following this decline, the population has failed to recover, and the contemporary population consists of approximately 300 adult individuals [18]. The failure to recover could be related to the small population size, but also changes in the alpine ecosystems causing expansion of the red fox (*Vulpes vulpes*) as well as fading cycles of small rodents [19].

Previous studies on mitochondrial DNA variation in the arctic fox have indicated a loss of unique genetic variants as the species' range contracted at the end of the last glaciation, as well as a marked loss of genetic diversity in connection with the human-induced bottleneck 100 years ago [10,20]. These studies have been based on short (less than 300 bp) DNA sequences from the mitochondrial control region, and the results may thus not be representative of the overall mitogenomic diversity. However, recent advances in DNA sequencing technology have now made it possible to recover mitogenome-scale datasets from ancient specimens at a reasonably low cost and effort [21,22].

The aim of this study was to examine the consequences of both the end-Pleistocene range contraction and more recent human persecution on the mitogenome diversity in the arctic fox. A better understanding of these past processes could help refine our ability to predict the impact of ongoing changes in climate and demographic bottlenecks. To investigate these questions, we generated a temporally sampled mitogenomic dataset that spanned from the Late Pleistocene to the present day, and included samples from mid-latitude Europe, northeastern Russia and Scandinavia. Because previous results have suggested that the Late Pleistocene mid-latitude European populations did not contribute to the present-day mitochondrial DNA gene pool [10], we expected to find relatively high sequence divergence between the ancient samples from mid-latitude Europe and present-day Scandinavia. By contrast, because the Scandinavian population has been proposed to originate from a postglacial expansion from Beringia [10], we expected low sequence divergence (i.e. direct ancestry) between Pleistocene samples from northeastern Russia and present-day Scandinavia. Additionally, we expected that a mitogenomic dataset would permit a more accurate assessment of the loss of genetic diversity and changes in effective population size during the last 100 years, compared to previous studies on shorter mitochondrial DNA sequences.

## 2. Material and methods

### (a) Samples and data generation

We sampled 30 historical Scandinavian specimens, collected between 1845 and 1927, and 26 Late Pleistocene Eurasian remains that ranged in age from 30 000 to 12 000 years before present (BP) (electronic supplementary material, tables S1 and S2). The samples comprised mainly arctic fox teeth and bones, and also one complete frozen carcass (sample IN18-002; electronic supplementary material, figure S4). The frozen carcass was recovered from permafrost deposits in Belaya Gora (Yakutia, Russia), and was sent for dating at the Oxford Radiocarbon Accelerator Unit.

DNA extractions from the historical as well as the comparatively well-preserved permafrost Pleistocene bone and tooth samples were conducted following the protocol described in Ersmark *et al*. [23]. The non-permafrost Pleistocene samples were extracted using a method optimized for highly degraded samples [24], and the skin and claw samples were extracted using a method optimized to digest keratin-rich tissues [25].

For the ancient and historical samples, DNA libraries were constructed according to the protocol by Meyer and Kircher [26], but with an additional step before the blunt-end repair. Here, the samples were incubated with the uracil-specific excision reagent (USER) enzyme to remove DNA damage that is characteristic for historical and ancient samples. The DNA libraries were subsequently amplified through polymerase chain reaction (PCR) (using 12 to 16 cycles) together with sample-specific indexing primers. To purify and simultaneously conduct a size selection of the post-amplified libraries, Agencourt AMPure XP beads were used. The concentration of each library was measured on a Bioanalyzer2100 (Agilent) and the libraries were subsequently pooled in equimolar ratios before being sent to the Science for Life Laboratories (Sci*Life*Lab) in Stockholm for paired-end shotgun sequencing on the Illumina HiSeq 2500 High Output V4 platform with a 2 × 125 bp setup.

To minimize the risk of contamination, all pre-PCR work on the ancient samples was done in a dedicated ancient DNA laboratory at the Swedish Museum of Natural History (NRM) following standard practices [27]. Similarly, the work on historical samples was done in a second laboratory dedicated to work on degraded historical DNA. Both of these laboratories are physically separated from the modern DNA and post-PCR laboratories.

In addition to the Pleistocene and historical samples, raw genomic data were generated from 21 modern Scandinavian samples, seven farm fox samples (from fur farms), two modern Russian samples and one modern Canadian sample (electronic supplementary material, table S3). The rationale for including farm foxes was partly that these originate from, and thus represent, wild populations in Svalbard and North America. In addition, an earlier study had found a control region haplotype shared between farm and historical Scandinavian foxes [20], and we were interested in resolving whether this result held up when extending the data to a mitogenomic dataset. DNA from 20 samples was extracted at NRM using the Thermo Scientific KingFisher Cell and Tissue DNA kit, while DNA from 11 samples was extracted at the Norwegian Institute for Nature Research (NINA), Trondheim, using the Qiagen DNeasy Blood & Tissue Kit. The samples were subsequently sent to Sci*Life*Lab, Stockholm, and the Genomics Core Facility at the NTNU, Trondheim, for Illumina TruSeq PCR-free library construction and deep-sequencing on the Illumina HiSeq X and Illumina HiSeq 4000 platforms, respectively.

### (b) Data processing

The resulting sequence data were processed following the approach in Pecnerova *et al*. [21]. In brief, the sequencing reads were trimmed of adapters and paired reads were merged using the software SeqPrep 2.2 (http://github.com/jstjohn/SeqPrep). The merged reads were then mapped to a reference mitochondrial genome of the arctic fox (GenBank accession no. NC_026529.1), using the BWA aln algorithm [28]. Next, the alignments were converted from SAM to BAM format, coordinate sorted, indexed, and PCR duplicates were removed using SAMtools [29] and a custom script [21]. Mapped reads were subsequently extracted and imported into Geneious 8.1.6 [30]. Consensus sequences were called for all samples separately, using the majority call rule with a minimum coverage of at least three independent reads. To estimate endogenous DNA content in the Pleistocene and historical samples, the raw reads from each sample were mapped to an internally generated de novo assembly of the arctic fox genome (J. von Seth, K. Norén, A. Angerbjörn, L. Dalén 2016, unpublished data), and the proportion of reads that mapped to the reference genome prior to duplicate removal was estimated with Qualimap 2 [31].

The mapped mitochondrial reads were inspected manually in Geneious and a BLAST-search was conducted of all reads that contributed to a mutation, in order to identify potentially falsely mapped nuclear mitochondrial segments (numts). Out of a total of 119 segregating sites, only one modern sample contained a mutation that appeared to be derived from a numt (at site 55; sample 5963; electronic supplementary material, table S3). This site was therefore deleted in all samples. Ten samples showed a heterozygote pattern within a tandem repeat region in the D-loop. All samples had equal ratios of G and C in the 13th position of the 14 bp tandem repeat. Owing to this, the tandem repeat region (252 bp) was excluded from the subsequent data analyses.

### (c) Computational analyses

A Bayesian phylogeny was reconstructed in BEAST 2.0 [32], using date calibrated tips, the HKY + I substitution model and a uniform prior of the mutation rate of the mitochondrial genome set to 6.1 × 10^−8^ to 1.0 × 10^−7^ site^−1^ yr^−1^ (calculated for grey wolf, *Canis lupus* [33]). The substitution model was selected according to the Bayesian information criterion in JModeltest2 [34]. We investigated genetic variability in arctic foxes between time periods, using a median-joining network created in PopArt 1.7 [35]. We also estimated population genetic diversity parameters, such as haplotype and nucleotide diversity, with DnaSP v5 [36]. Haplotype accumulation curves were constructed through random permutation subsampling without replacement using the functions haploAccum() and plot.haploAccum() supplied in the SPIDER package in R [37]. We also used an approximate Bayesian computation (ABC) approach [38,39] to estimate changes in female effective population size (*N_ef_*) over the last 150 years. Our model included a single human-induced decline occurring some 90–120 years ago, a generation time of 3 years and mean rate ranging from 1.85 × 10^−7^ to 3.00 × 10^−7^ substitutions generation^−1^ (electronic supplementary material, table S4; [33]). In the model, we set the sampling times for modern and historical samples at *t* = 0 generations and *t* = 40 generations in the past, respectively. We used seven summary statistics and estimated posterior parameter distributions by retaining the 10 000 datasets (1%) with the smallest Euclidean distances to the observed dataset.

## 3. Results

### (a) Data processing and final datasets

Eleven out of the 26 Pleistocene samples and 20 out of the 30 historical samples had an endogenous DNA content above 10% (electronic supplementary material, tables S1 and S2). Among the non-permafrost Pleistocene samples, originating from mid-latitude Europe and Russia, one-third of the samples had endogenous DNA content above 10%. The average endogenous DNA content for the Pleistocene permafrost samples was 72% (electronic supplementary material, table S2). Samples with endogenous DNA content exceeding 10% are generally considered highly suitable for whole-genome sequencing, and these results, therefore, suggest that a large proportion of our samples could be used in future studies of autosomal genetic diversity.

The complete arctic fox carcass from Belaya Gora was dated to 18 350 ± 130 radiocarbon years BP (radiocarbon laboratory reference OxA-38194), and was calibrated using the international calibration curve ‘IntCal13’ [40,41], to 22 468–21 887 (95.4%) cal BP.

**Figure 1.**
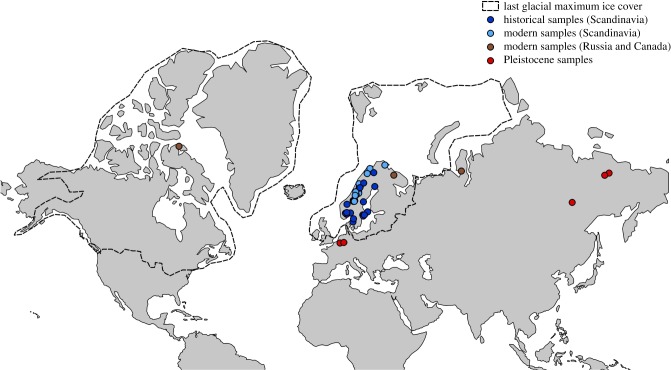
Geographical distribution of the samples used in this study. Light and dark blue dots represent modern and historical samples, respectively. Red dots represent Pleistocene samples and brown dots represent modern samples from Canada and Russia. Striped black lines represent the ice cover during the last glacial maximum [42]. (Online version in colour.)

The low-coverage sequencing, variable endogenous DNA content and high clonality in the Pleistocene samples did not permit reconstruction of entire mitogenomes from all the samples. Thus, in order to maximize the number of Pleistocene samples included in the comparisons, we compiled a reduced alignment comprising 3954 nucleotide sites (at greater than 3x coverage). However, to obtain an even more accurate estimate of the loss of mitogenomic diversity during the last 100 years, we also constructed a second alignment that comprised 13 224 sites (at greater than 3x coverage), which excluded all Pleistocene samples. Taken together, both datasets comprised five Pleistocene samples (two from Western Europe and three from Beringia), 16 historical samples, and all 24 modern samples (including the modern samples from Russia and Canada), plus seven modern farm fox samples (figure 1).

### (b) Phylogeny and median-joining network

**Figure 2.**
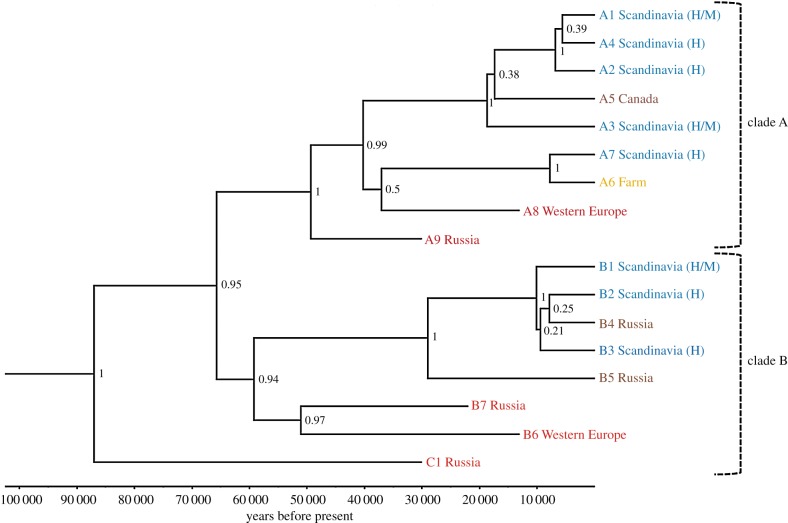
Phylogeny of arctic fox haplotypes based on 3954 bp of the mitogenome. Haplotypes are labelled after clade and location, where Scandinavian haplotypes found in historical samples are indicated with an H and modern samples with an M. Posterior values for node support are shown for each node. (Online version in colour.)

**Figure 3.**
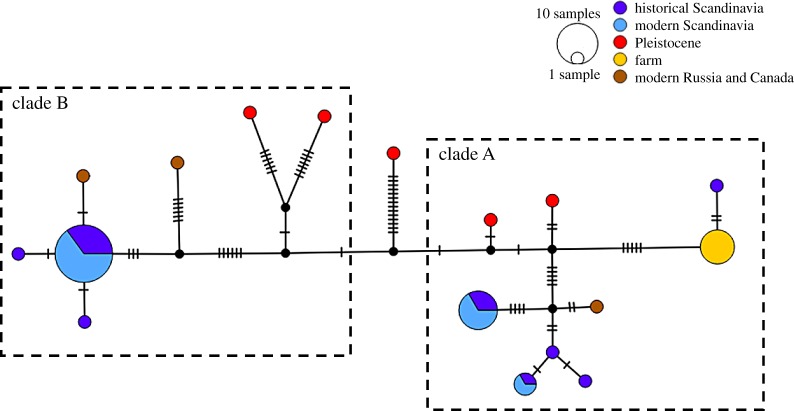
Median-joining haplotype network based on 3954 bp of the mitogenome. Small crossing bars represent mutations between haplotypes. Colours correspond to sample population. Dotted lines indicate clade A and clade B as identified in the BEAST analysis (figure 2). (Online version in colour.)

The Bayesian phylogeny (figure 2) suggested that the most recent common female ancestor lived *ca* 87 000 years BP (mean: 87 038 BP; 95% highest posterior density (HPD) = 44 571–153 339 BP). The phylogeny also revealed that all present-day, and most Late Pleistocene, mitogenome haplotypes can be divided into two separate clades with a posterior probability node support of 0.95. The split into these two clades was estimated to have occurred *ca* 66 000 years BP (mean: 65 756 BP; 95% HPD = 33 196–122 310 BP). There was no apparent phylogeographic structure within or between the clades. Clade A included historical and modern Scandinavian, Canadian and farm arctic foxes, and Pleistocene Russian and Pleistocene western European arctic foxes. Interestingly, the mitogenomic dataset allowed us to demonstrate that historical Scandinavian and farm foxes do not share the same haplotype, meaning that free-ranging present-day foxes with the farm fox haplotype now can be conclusively assigned as having farm fox ancestry (see [43]). Clade B included modern and historical Scandinavian, modern Russian, Pleistocene Russian and Pleistocene western European arctic foxes (figure 2). The median-joining haplotype network illustrates the separate clades, with the Pleistocene Russian haplotype C1 placed in an intermediary position between the two clades (figure 3).

### (c) Changes in mitogenomic variation and *N*_ef_ in Scandinavia

**Figure 4.**
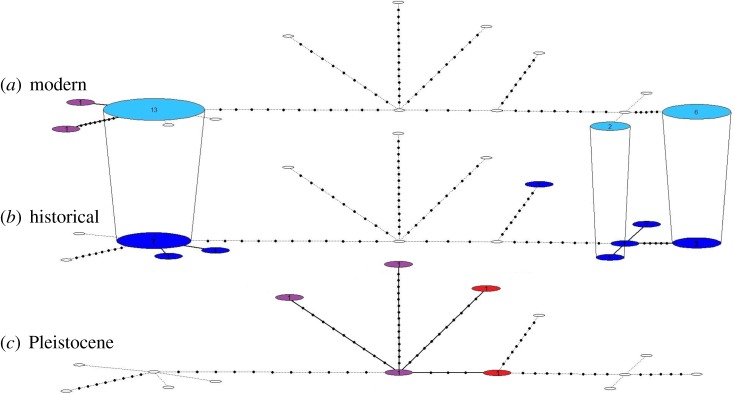
Temporal haplotype network of samples from Eurasia based on 3954 bp of the mitogenome. Circles represent haplotypes and numbers represent sample sizes. (*a*) Haplotypes found in modern-day Eurasia. (*b*) Haplotypes found in the historical Scandinavian population. (*c*) Pleistocene haplotypes. Mid-latitude European samples are shown in red, Russian samples are shown in purple, whereas Scandinavian historical and modern samples are shown in dark and light blue, respectively. Empty circles represent haplotypes absent in the given time period. Haplotypes found in multiple time periods are connected with vertical lines. (Online version in colour.)

**Figure 5.**
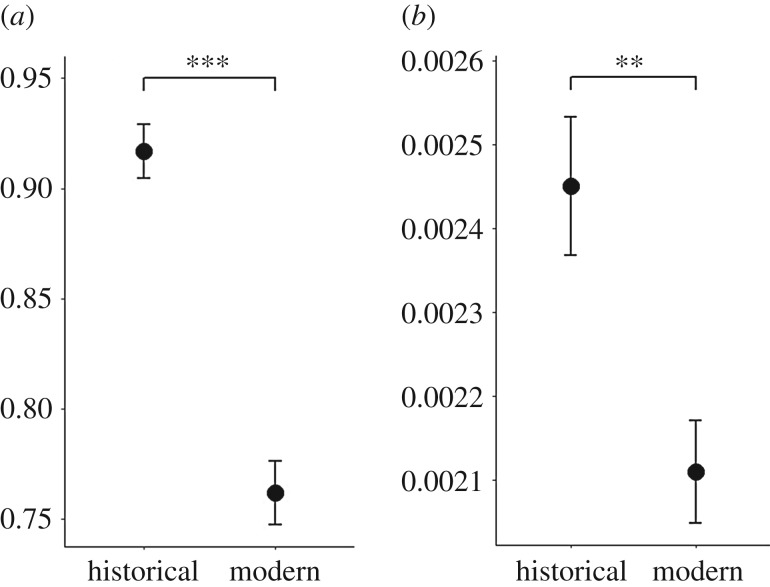
Haplotype diversity (*a*) and nucleotide diversity (*b*) in the historical and contemporary Scandinavian arctic fox populations. Asterisks indicate significant differences among time periods, where **p < 0.01 and ***p < 0.001. Error bars depict standard error.

In Scandinavia, we identified 10 and five haplotypes in the historical and modern samples, respectively, in the alignment comprising 13 224 bp of the mitochondrial genome (electronic supplementary material, figures S1 and S5). Using the shorter alignment including the Pleistocene samples, we found eight and three haplotypes in the historical and modern populations, respectively (figures 3 and 4). The loss of haplotypes in the Scandinavian arctic fox population also resulted in a significant decline in nucleotide diversity (*π* ± s.e.) (0.00245 ± 0.00008 versus 0.00211 ± 0.00006; *t*_37_ = 3.39, *p* < 0.01) and haplotype diversity (Hd ± s.e.) (0.917 ± 0.012 versus 0.762 ± 0.014; *t*_37_ = 7.85, *p* < 0.001) (figure 5). In the modern population, three haplotypes were shared with the historical population, while two haplotypes were private: A10 was found in two samples from Lierne and two samples from Borga, and B9 was found in two samples from Vindelfjällen and two samples from Borga (electronic supplementary material, figure S1 and table S3). The ABC analysis supported an almost 20-fold reduction in female effective population size with *N_ef_* declining from approximately 3650 to approximately 220 over the last 150 years (electronic supplementary material, table S4).

## 4. Discussion

### (a) Consequences of past climate change

In contrast to earlier studies, the recovery of a mitogenomic dataset allowed us to generate a robust phylogeny and an assessment of divergence times among lineages. This showed that the most recent female common ancestor of the arctic fox probably lived less than 100 000 BP (figure 2). This is consistent with previous suggestions that the arctic fox went through a bottleneck during the warm Eemian interglacial [44], which had its peak *ca* 125 000 BP with average temperatures in the Arctic about 2–5 degrees warmer than today [45]. Our results also showed that the mitogenomes in present-day arctic foxes can be divided into two distinct clades, which diversified approximately 60 000 years ago. The reason for this diversification into two clades remains to be investigated, but could be related to the onset of rapid stadial-interstadial fluctuations in temperature during Marine Isotope Stage 3 [46]. Moreover, it is noteworthy that there seems to have been no clear phylogeographical structure during at least the last 30 000 years, which probably is a result of the high dispersal capability of the arctic fox (see also [44]). Even though the large ice sheets covering much of northern Europe and North America probably were a poor habitat for arctic foxes, these animals are capable of long movements over the polar pack ice [47]. They would therefore still have been able to disperse and admix throughout the circumpolar area during the Late Pleistocene [48].

The two ancient western European samples, which are approximately 12 000 years old, were not closely related to any of the modern or historical samples (figures 2 and 4). While these two samples do not by themselves permit a comprehensive assessment of the mid-latitude European population's genetic diversity, when combined with the results from Dalén *et al*. [10] they reinforce the conclusion that the mid-latitude European population did not contribute to the present-day mitochondrial diversity in the arctic fox. Together, these studies therefore support the hypothesis that the mid-latitude European arctic fox population went extinct at the end of the last glaciation, and thus that the species' range contraction was driven by local extinction events rather than postglacial habitat tracking.

An alternative hypothesis is that the mid-European population did in fact recolonize the Scandinavian Peninsula during the early Holocene, when previously glaciated areas became suitable habitats, but that it subsequently went extinct later in the Holocene. Subfossil records of arctic foxes in Norway suggest a gap in the fossil record between *ca* 9000 BP and *ca* 5000 BP, coinciding with a warm period in the mid-Holocene. It has been hypothesized that the arctic fox disappeared from the Scandinavian Peninsula during this period, and that another recolonization event occurred around 5000 BP [49]. Although this scenario would also imply that the arctic foxes failed to track the climate-induced changes in habitat, it is thus uncertain whether this occurred during the Pleistocene/Holocene transition or during the subsequent Holocene warm peak. To examine this further, genetic data from postglacial Scandinavian samples that are older than 9000 BP would be needed.

Intriguingly, and contrary to our expectations, the Pleistocene Russian samples from Beringia were also genetically distinct from the present-day population in Scandinavia (figures 2 and 4). It should be noted that the small sample size (*n* = 3) does not allow us to exclude that the Scandinavian population originates from the Beringian Late Pleistocene population. However, although speculative, these preliminary findings could suggest that the postglacial recolonization of Scandinavia originated from another, yet unsampled, Late Pleistocene population of arctic foxes, which in turn could also imply that a local extinction/recolonization event may have taken place in Beringia at the end of the last glaciation. This hypothesis is consistent with earlier findings of end-Pleistocene population turnover in several other Arctic taxa in Beringia [5,6].

### (b) Consequences of recent human persecution

Our results show that the Scandinavian arctic fox population lost at least 50% of its mitochondrial haplotypes as a result of the demographic bottleneck that started at the turn of the twentieth century (figure 4; electronic supplementary material figure S2). Moreover, compared to previous studies [20], the higher resolution of the mitogenomic sequences allowed for better estimations of both haplotype and nucleotide diversity, revealing previously unknown significant losses following the bottleneck (figure 5). The coalescent simulations coupled with ABC analyses suggested an approximately 20-fold decline in *N_ef_*, which is roughly in line with expectations based on historical records [16,17].

We found five previously unknown haplotypes, three in the historical population and two in the contemporary population. The two new haplotypes found in the modern population were not shared with the historical population. A possible explanation for these private haplotypes in the modern population could be insufficient sampling in the historical population. Haplotype accumulation curves suggested that the asymptote for the modern population was reached at five haplotypes, while the haplotype accumulation curve for the historical population failed to reach the asymptote (electronic supplementary material, figure S2). Further sampling might thus identify these two modern haplotypes (A10 and B9; electronic supplementary material, figure S1 and table S3) in historical samples. It is also possible that the private haplotypes are the result of random mutations. However, taking the mutation rate of the mitochondrial genome into account, it seems highly unlikely that this would have happened in two individuals in less than 150 years. A third possible explanation would be recent immigration events from the population in Russia, which would be in line with results from previous studies [20,50].

Finding only 10 haplotypes in the pre-bottleneck population may seem relatively low, even when taking into account that further sampling probably would have revealed additional haplotypes (electronic supplementary material, figure S2). Such a low number of haplotypes could be a consequence of Scandinavia being an isolated peninsula with a recent glaciation history. Populations on peninsulas generally have less genetic diversity than mainland populations [51,52]. Moreover, populations in previously glaciated regions are expected to have a reduced genetic diversity because there has not been enough time since colonization to reach mutation-drift equilibrium (e.g. [53]). Indeed, results from studies on other historical Scandinavian carnivore populations (pre-dating recent bottlenecks) have shown that the Scandinavian grey wolf, brown bear (*Ursus arctos*), lynx (*Lynx lynx*) and wolverine (*Gulo gulo*) also have relatively low levels of mitochondrial diversity [54–57]. Thus, the relatively recent colonization of the region, which started with the contraction of the Scandinavian ice sheet at the end of the last glaciation, combined with a small number of founders and comparative isolation of Scandinavia as a peninsula, could explain the low diversity of the pre-bottleneck Scandinavian arctic fox population.

### (c) Conservation implications

The distribution of the Arctic biota decreased considerably during the transition from the last glaciation to the present interglacial. Our results suggest that a contraction in available habitat (during the Pleistocene/Holocene transition or the mid-Holocene) caused local subpopulations to become extinct, which in turn led to losses of unique genetic diversity. This implies that the genetic diversity in many Arctic species probably was reduced even before the impact of human activities that accelerated during the industrial revolution. This might have affected the adaptive potential in the arctic fox as well as other cold-adapted species, thus increasing the impact of subsequent anthropogenic activities. Furthermore, there are suggestions that climate change has again started to affect the Scandinavian arctic fox population in recent decades, through an expansion and ensuing interspecific competition from red foxes that have moved into mountain areas previously inhabited by the arctic fox [58]. Assuming that these consecutive losses of mitogenomic diversity, owing to climate change and anthropogenic impact, also reflect changes in autosomal genetic diversity, this would imply that the arctic fox might be particularly vulnerable to the future challenges entailed by climate change.

Importantly, these findings indicate that climate-driven range contractions do not simply lead to a reduction in population size, but also that local populations become extinct without contributing to the gene pool of populations further north. This implies that future climate-induced range contractions may lead to larger losses of genetic diversity than predicted from simplistic models based solely on population size. Moreover, unique genetic variants (e.g. local adaptations) in southern populations will probably be lost as these populations become extinct. At a broader level, such a lack of habitat tracking needs to be accounted for in climate-based models that predict future extinction rates [59], and raises a concern about the efficiency of conservation corridors [60] designed to enable species to move into new habitats as the climate changes.

In the case of the Scandinavian arctic fox, this population is currently fragmented into several relatively isolated subpopulations along the Scandes mountain range [50]. Our results suggest that future changes in tree-line extent and red fox distribution will probably lead to a northwards contraction of the arctic fox's distribution, and thus a loss of any genes unique to the southernmost subpopulations.

## Supplementary Material

Supplementary items

## Supplementary Material

Supplementary tables

## Data accessibility

The mtDNA data are available in the form of BAM files, which can be downloaded at the European Nucleotide Archive (EA) under accession number PRJEB34472.
