# Supplementary material for: Consequences of past climate change and recent human persecution on mitogenomic diversity in the arctic fox
Source: Philos Trans R Soc Lond B Biol Sci. 2019 Nov 4;374(1788):20190212. doi: 10.1098/rstb.2019.0212 (PMC6863501; doi:10.1098/rstb.2019.0212)
Supplement: Supplementary items [file rstb20190212supp1.pdf]

Supplementary information

Consequences of past climate change and recent human persecution on mitogenomic diversity in the arctic fox

Petter Larsson, Johanna von Seth, Ingerid J. Hagen, Anders Götherström, Semyon Androsov, Mietje Germonpré, Nora Bergfeldt, Sergey Fedorov, Nina E. Eide, Natalia Sokolova, Dominique Berteaux, Anders Angerbjörn, Øystein Flagstad, Valeri Plotnikov, Karin Norén, David Díez-del-Molino, Nicolas Dussex, David W. G. Stanton, Love Dalén.

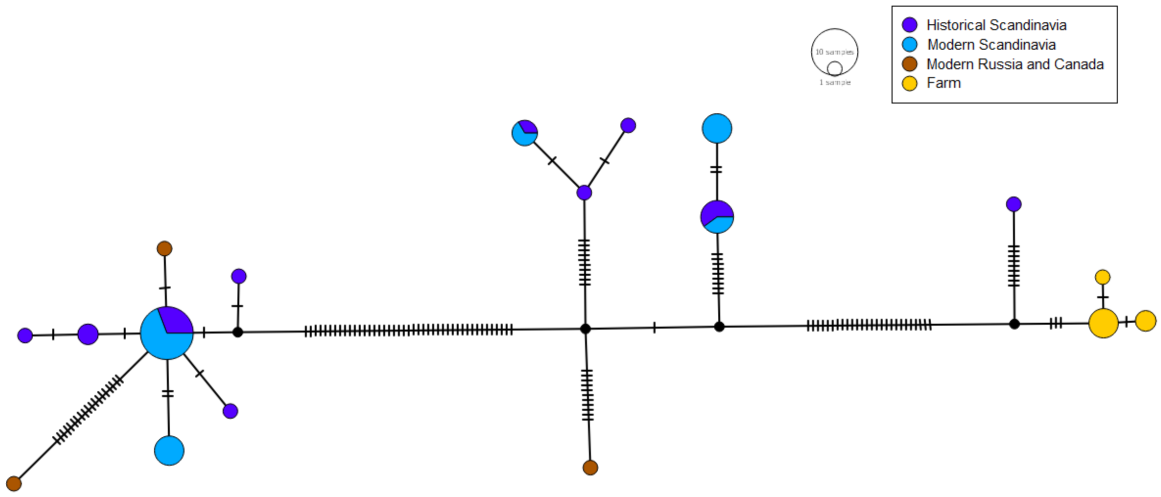

Figure S1. Median-joining haplotype network based on 13,224 bp of the mitochondrial genome. Historical and modern Scandinavian arctic fox populations are colored in dark blue and light blue respectively. Farm fox haplotypes are colored in yellow and modern samples from Canada and Russia are colored in brown.

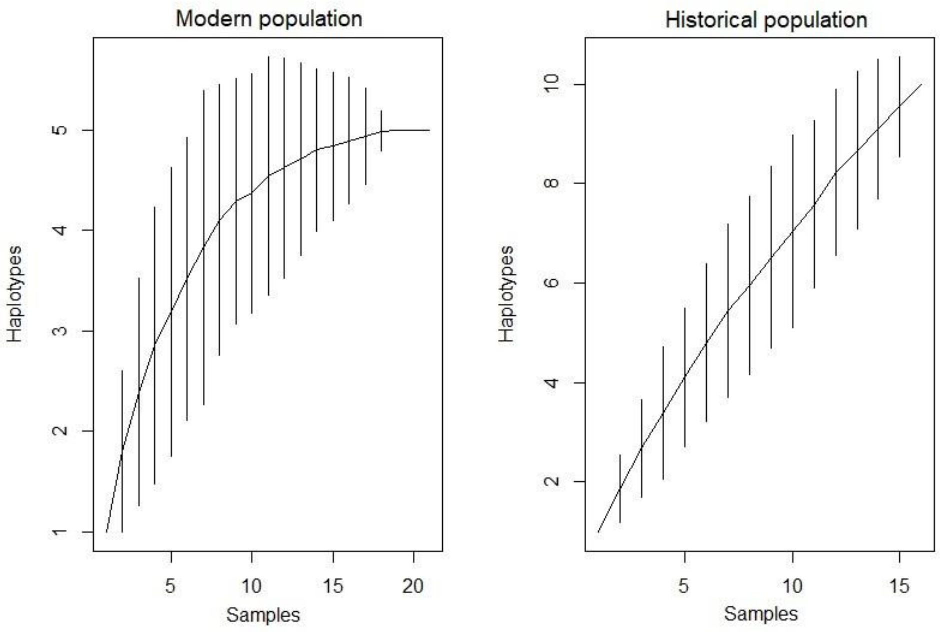

Figure S2. Haplotype accumulation curves of the modern (n=21, left) and historical (n=16, right) Scandinavian arctic fox population. The mean (cumulative line) and standard errors (vertical lines) were calculated though 100 random permutations.

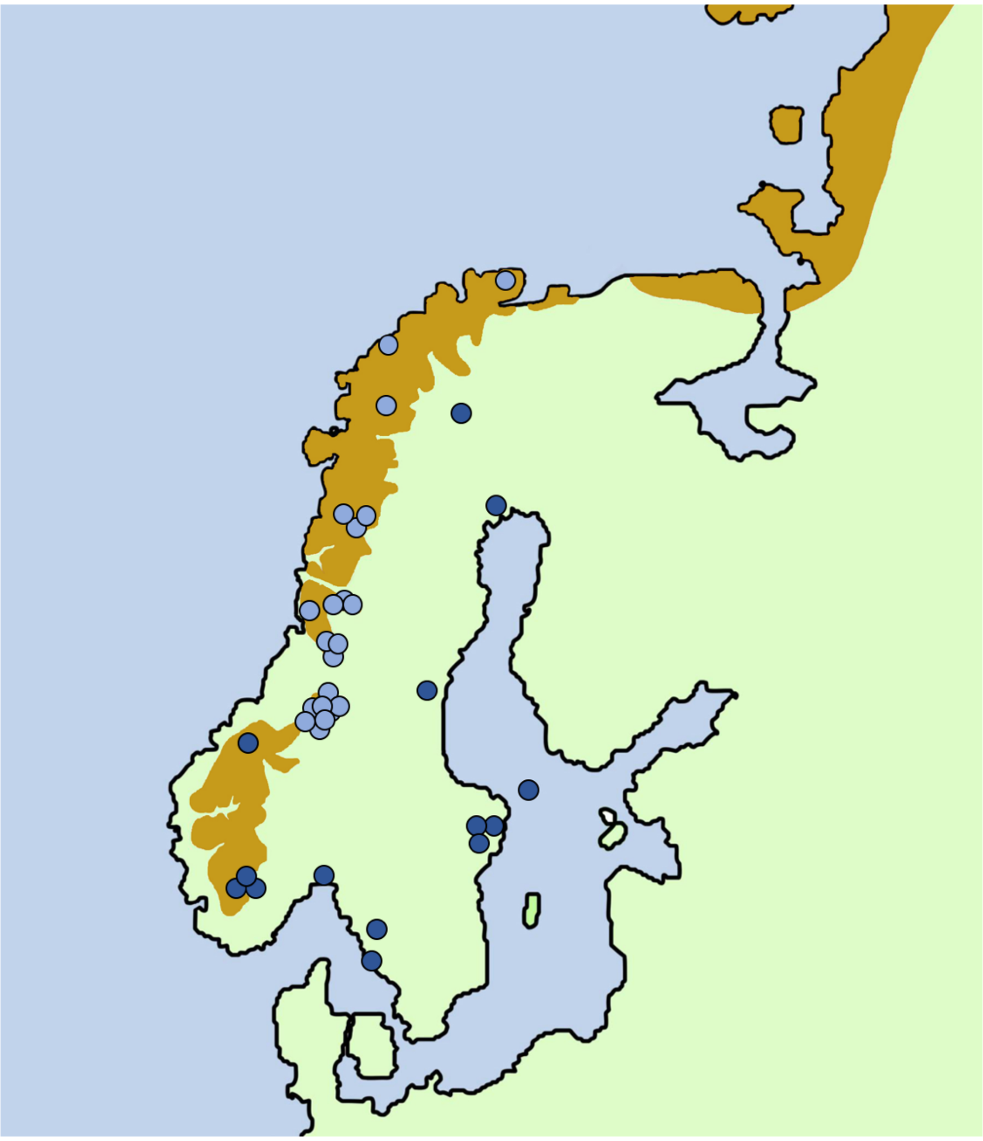

Figure S3. Geographical distribution of the Scandinavian samples used in this study. Present-day arctic fox habitats are shown in brown. Dark and light blue dots represent historical and modern samples, respectively.

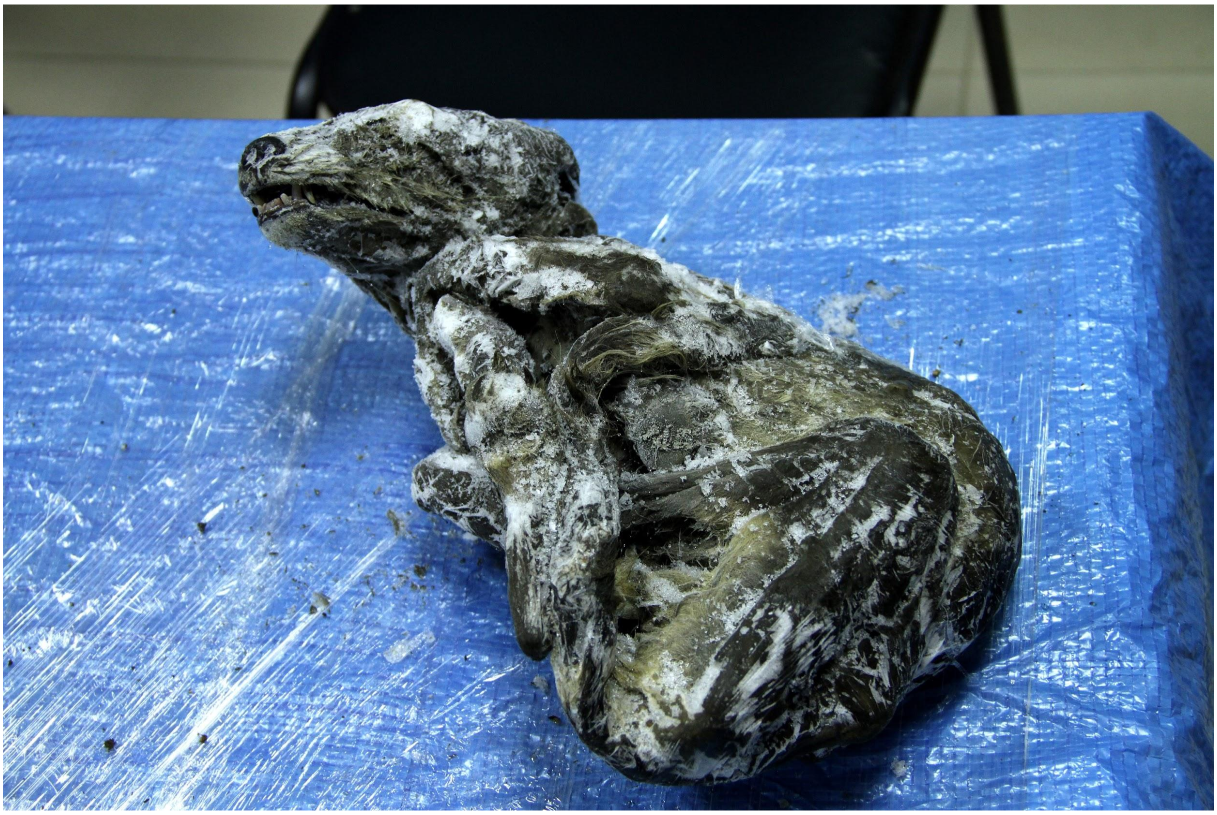

Figure S4. Frozen carcass of an arctic fox discovered in permafrost deposits from Belaya Gora in northeastern Siberia, and radiocarbon dated to 18,350 ±130 radiocarbon years before present.

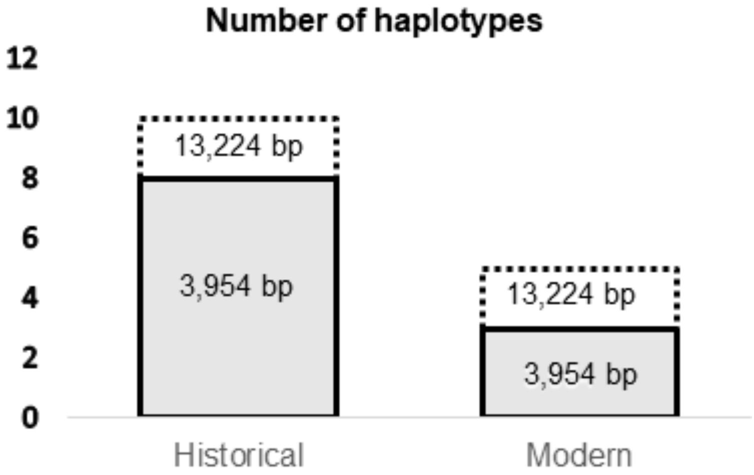

Figure S5. Number of mitogenomic haplotypes detected in the historical and modern population. Solid bars show the number of haplotypes based on a 3,954 bp alignment, whereas unfilled bars show haplotype numbers using a 13,224 bp alignment.

Table S4. Prior and posterior distributions, including the highest posterior distributions (HPD), for a model of decline over the last 90-120 years. The timing of the decline is in number of generations assuming a generation time of 3 years (Koblmuller et. al 2016).

Parameter	Prior distribution	Posterior mode	HPD 5%	HPD 95%
Modern Nef	Uniform [10 - 10^3]	220	106	755
Historical Nef	Uniform [10^3 - 5 × 10^5]	3,650	2,100	9,130
$t_{hum\text{-}bot}$	Uniform [30 - 40]	39	30	40
μ rate	Uniform [1.85 × 10^{-7} –3.00 × 10^{-7}]	2.32 × 10^{-7}	1.88 × 10^{-7}	2.89 × 10^{-7}
